# Morphological and Histological Features of the Vomeronasal Organ in African Pygmy Hedgehog (*Atelerix albiventris*)

**DOI:** 10.3390/ani11051462

**Published:** 2021-05-19

**Authors:** Daisuke Kondoh, Yusuke Tanaka, Yusuke K. Kawai, Takayuki Mineshige, Kenichi Watanabe, Yoshiyasu Kobayashi

**Affiliations:** Department of Veterinary Medicine, Obihiro University of Agriculture and Veterinary Medicine, Nishi 2-11 Inada-cho, Obihiro 080-8555, Japan; dondonobihiro@outlook.jp (Y.T.); ykawai@obihiro.ac.jp (Y.K.K.); mineshige@obihiro.ac.jp (T.M.); knabe@obihiro.ac.jp (K.W.); kyoshi@obihiro.ac.jp (Y.K.)

**Keywords:** chemosensory system, exocrine glands, hedgehogs, kairomones, olfaction, pheromones, serous secretion, vomeronasal organ

## Abstract

**Simple Summary:**

Hedgehogs have a sensitive olfaction, but little is known about their vomeronasal organ, which detects specific chemicals such as pheromones. This is the first study to reveal the morphological and histological features of the vomeronasal organ in the African pygmy hedgehog. Notably, unlike other mammals, the hedgehog has a large, well-developed serous gland in the vomeronasal organ. This gland seems to allow flushing out odorous substances from the vomeronasal organ and might be favorable for subsequent stimulus reception.

**Abstract:**

The vomeronasal organ (VNO) detects specific chemicals such as pheromones and kairomones. Hedgehogs (Eulipotyphla: Erinaceidae) have a well-developed accessory olfactory bulb that receives projections from the VNO, but little is known about the hedgehog VNO. Here, we studied the histological features of the VNO in five individual African pygmy hedgehogs by hematoxylin-eosin, periodic acid-Schiff, and Alcian blue stains. The hedgehog VNO comprises a hyaline cartilage capsule, soft tissue and epithelial lumen, and it branches from the site just before the incisive duct opening into the nasal cavity. The soft tissues contain several small mucous (or mucoserous) glands and a large serous gland, and many venous sinuses all around the lumen. The VNO lumen is round to oval throughout the hedgehog VNO, and the sensory epithelium lines almost the entire rostral part and medial wall of the middle part. These findings indicate that the VNO is functional and plays an important role in the hedgehog. Notably, the VNO apparently has a characteristic flushing mechanism with serous secretions like those of gustatory glands, which the hedgehog might frequently use to recognize the external environment.

## 1. Introduction

Most mammals detect environmental chemicals using main olfactory and vomeronasal systems. The vomeronasal organ (VNO) detects specific chemicals that benefit animals, such as pheromones and kairomones, which are respectively released by the same and other species [1].

The mammalian VNO composes a cartilage (or bone) capsule, highly glandular and vascular soft tissue, and an epithelial lumen with a blind end at the caudal side, and a species-specific rostral orifice [2,3]. The lumen of the VNO in most mammals is generally crescent-shaped, and the medial and lateral regions of the lumen are lined by sensory and non-sensory epithelia, respectively [3]. Mucous (or mucoserous) glands associated with the VNO secret into the lumen [3] to form a mucous layer of the sensory epithelium. Odorant substances are taken in and out of the lumen of the VNO by the expansion and contraction of blood vessels, namely venous sinuses [4,5,6], and are detected after dissolution in mucous fluids covering the sensory epithelium [3]. However, the morphology of the VNO varies according to species because it reflects their ecological features [7].

Hedgehogs (family Erinaceidae; order Eulipotyphla) are most closely related to shrews (family Soricidae) [8]. Wild African pygmy hedgehogs (*Atelerix albiventris* (Wagner, 1841)) inhabit the savannah and steppe regions of central and eastern Africa and they are bred as pets. They have a sensitive olfactory system, and olfaction seems to play key roles in behavioral navigation, food and predator detection, and conspecific communication [9]. Hedgehogs occasionally exhibit the flehmen response that transfers substances into the VNO [9], and they have a large, well-developed, accessory olfactory bulb that is a primal central part of the vomeronasal system [10], indicating the importance of the VNO in the ecology of hedgehogs. In fact, Ivey et al. [9] described “the vomeronasal organ is also prominent”, but there are few grounds to directly support it because little is known about the hedgehog VNO, except via a recent brief report on long-eared hedgehogs (*Hemiechinus auritus*) [11]. To verify whether hedgehogs have a VNO that is useful for their ecology, the present study aimed to determine the detailed morphological and histological features of the VNO in African pygmy hedgehog and how its significance differs from that in other mammals.

## 2. Materials and Methods

### 2.1. Animals

A total of five African pygmy hedgehogs (*Atelerix albiventris* (Wagner, 1841)) were studied. The heads of three males (age 2.5–3.5 years) and two females (age 2 and 3.5 years), all mature, were obtained from sacrificed control animals of the other experiment not related to this study, approved by the Animal Care and Use Committee of Obihiro University of Agriculture and Veterinary Medicine (OUAVM) (approval number: 19–23). The heads were fixed in 15% neutral-buffered formalin for 3–7 days at room temperature, then used in the following procedures. The Animal Care and Use Committee of OUAVM was notified of the experimental protocol (notification numbers: 21–8), and the study proceeded according to Institutional Regulations on the Management and Operation of Animal Experiments.

### 2.2. Macroscopic Anatomy

Male and female heads (*n* = 1 each) were processed for macroscopic anatomical assessment of the VNO before the histological process described below, to confirm the location and length. The left lateral and basal walls composing the maxilla, palatine, and nasal bones were removed. Images of the left VNO attached to the nasal septum, in addition to the rostral part of the palate, were acquired using a STYLUS TG-3 Tough digital camera (Olympus, Tokyo, Japan).

### 2.3. Histological Process

The VNO was removed from the two heads used for macroscopic anatomy by inserting a scalpel between the vomeronasal cartilage and the nasal septum, and then embedded in paraffin using a standard procedure. The nasal parts of the other three heads were decalcified in Plank–Rychlo solution [12] for 3 h, then embedded in paraffin. Specimens were sequentially cut into 5-μm-thick transverse sections by using an LS-113 sliding microtome (Yamato Kohki Industrial Co., Ltd., Saitama, Japan) at 20-μm intervals. The sections were deparaffinized, stained with hematoxylin-eosin, periodic acid-Schiff (PAS) or Alcian blue (pH 2.5) [13], and then assessed using a Microphot-FX microscope (Nikon, Tokyo, Japan) equipped with a Digital Sight DS-5 M camera (Nikon, Tokyo, Japan).

## 3. Results

### 3.1. Morphological Features of Hedgehog VNO

**Figure 1:**
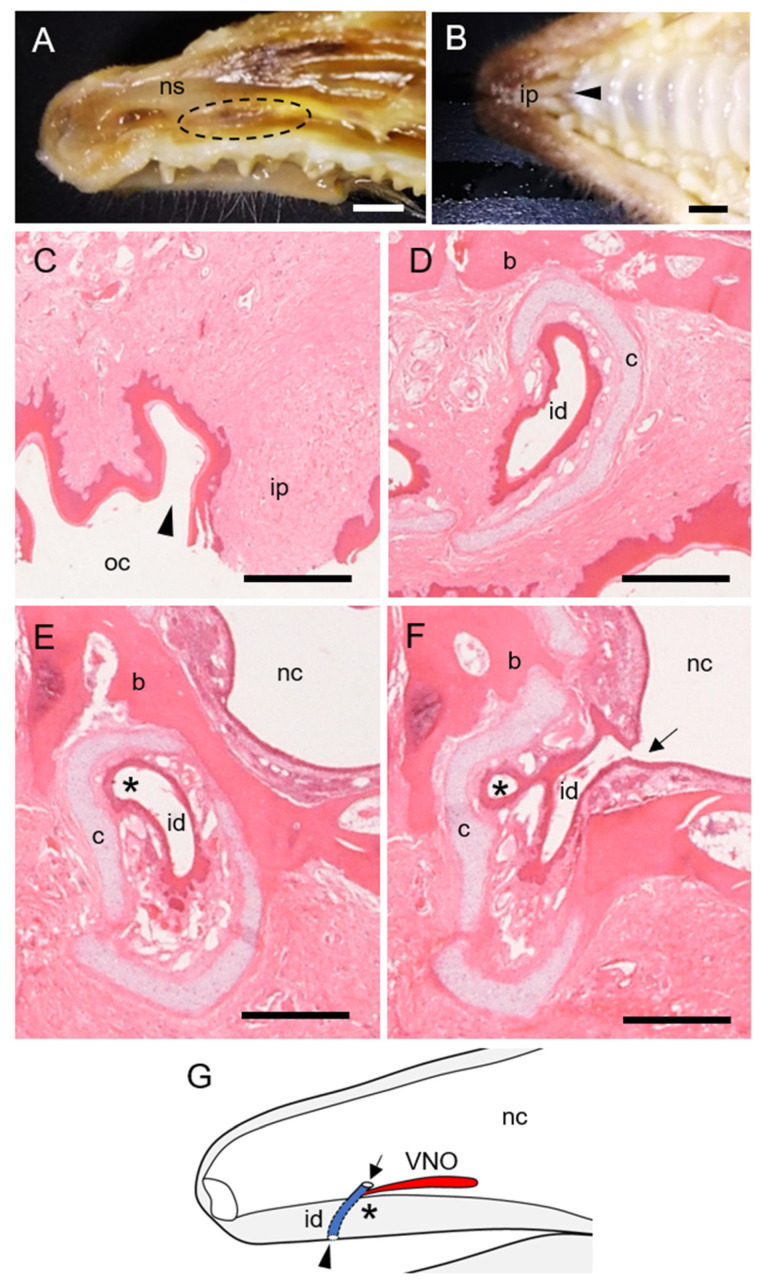
Hedgehog vomeronasal organ (VNO) location and opening. (**A**) Left lateral view of nasal septum (ns) after removing lateral walls. Dashed circle, VNO. (**B**) Ventral view of palate near incisive papilla (ip). Arrowhead, incisive ducts opening between the left and right incisive papillae. (**C**–**F**) Series of histological sections of incisive duct (id) from rostral (**C**) to caudal (**F**). Hematoxylin-eosin stain. Arrow and arrowhead, opening site of incisive duct into nasal (nc) and oral (oc) cavities, respectively; b, bone; c, hyaline cartilage. *, Opening of VNO. (**G**) Scheme of VNO opening. Bars, 5 mm (**A**,**B**) and 500 μm (**C**–**F**).

**Figure 2:**
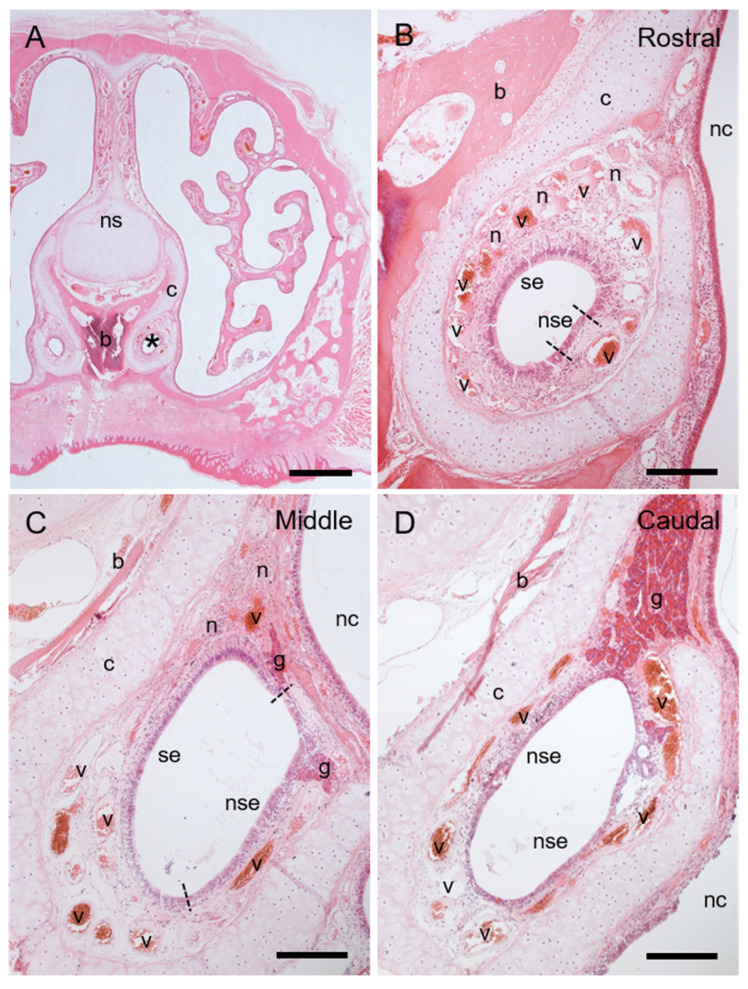
Histological components of hedgehog VNO. (**A**) Transversal image of rostral region of nasal cavity. Hematoxylin-eosin stain. * VNO attached to vomer bone (b) at base of nasal septum (ns). (**B**–**D**) Whole images of rostral (**B**), middle (**C**), and caudal (**D**) areas of VNO; b, bone; c, hyaline cartilage; g, glands; n, nerve bundles; nc, nasal cavity; nse, non-sensory epithelium; se, sensory epithelium; v, veins. Dashed lines, border between sensory and non-sensory epithelia. Bars, 1 mm (**A**) and 200 μm (**B**–**D**).

The VNO of the hedgehog was identified as a paired tubular organ (~1.0 cm in length) located at the basal part of the nasal septum. The VNO started at the first incisor in the longitudinal direction and ended near the first premolar (Figure 1A and Figure 2A). The incisive ducts were surrounded by hyaline cartilage and connected the nasal and oral cavities (Figure 1B–F). The VNO branched from a site just before the incisive duct opened into the nasal cavity (Figure 1E,F). Therefore, the opening of the VNO was closer to the nasal, than the oral cavity (Figure 1G). The VNO of the hedgehogs comprised hyaline cartilage, soft tissue, and epithelial lumen.

### 3.2. Cartilage and Soft Tissue Components of Hedgehog VNO

The vomeronasal cartilage continued from that covering the incisive duct (Figure 1D–F), and it assumed a J shape in cross sections throughout the VNO (Figure 2). The prominent structures in the soft tissue of the hedgehog VNO were veins, nerves, and secretory glands. Many large venous sinuses were located in the lamina propria all around the epithelial lumen throughout the entire length of the VNO (Figure 2B–D). Small thin nerve bundles were located in the lamina propria at the dorsomedial region of the rostral and middle areas (Figure 2B,C), and several thick bundles of vomeronasal nerves ran in the posterodorsal direction at the middle of the VNO (Figure 2C). 

**Figure 3:**
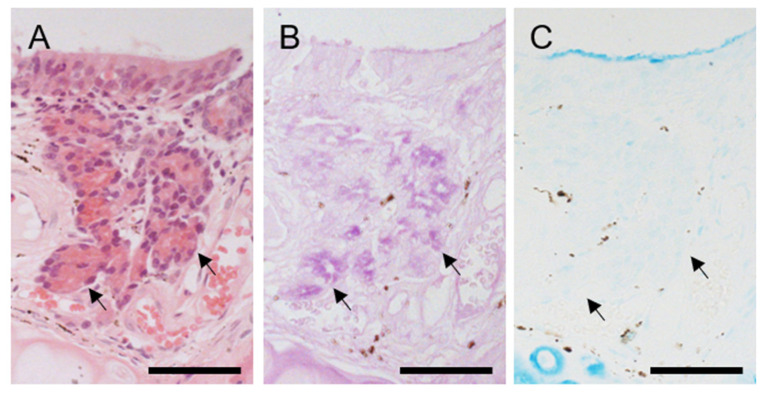
Histological features of glands in lateral region in middle of hedgehog VNO. (**A**) Hematoxylin-eosin stain. Each gland is composed of a small number of acini (arrows). (**B**) Periodic acid-Schiff (PAS) stain. These small vomeronasal glands are positive for PAS. (**C**) Alcian blue stain. These glands are negative for Alcian blue. Bars, 50 μm.

**Figure 4:**
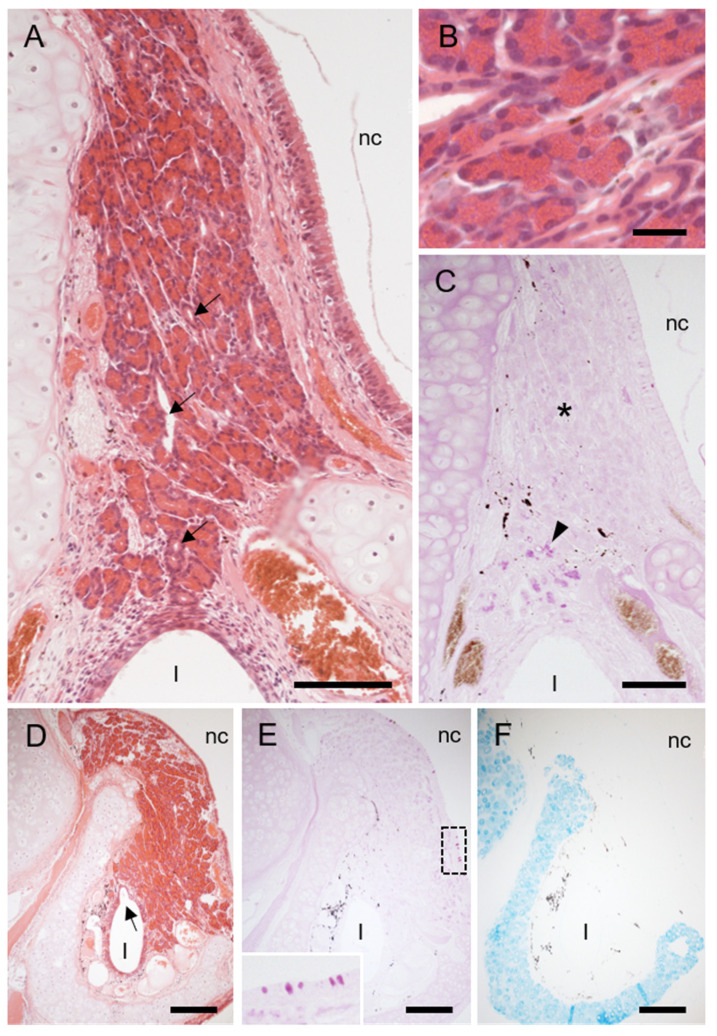
Histological features of large gland in dorsal region of caudal area of hedgehog VNO. (**A**) Hematoxylin-eosin stain. Arrows, significant secretory ducts. l, lumen of VNO; nc, nasal cavity. (**B**) Higher magnification of gland acini. Glandular cells contain many granules that are intensely stained with eosin. (**C**) Periodic acid-Schiff (PAS) stain. * PAS-negative acini. Arrowheads, a few acini near lumen of VNO positive for PAS. (**D**–**F**) Near caudal end of VNO lumen. (**D**) Hematoxylin-eosin stain. Arrow, secretory duct opening. (**E**) PAS stain. This gland is mostly negative for PAS. Dashed box, corresponding to insertion showing PAS-positive goblet cells in respiratory epithelium covering nasal cavity. (**F**) Alcian blue stain. This gland is negative for Alcian blue. Bars, 100 (**A**,**C**–**F**) and 20 (**B**) μm.

Two types of secretory glands were associated with the hedgehog VNO. Several small glands were located immediately below non-sensory epithelium in the lateral portion of the middle area (Figure 2C), and acini were stained positively for PAS but negatively for Alcian blue (Figure 3), indicating mucous (or mucoserous) glands containing neutral mucopolysaccharides. A large, concentrated gland was located in the dorsal part of the caudal area (Figure 2D) and continued posteriorly after the lumen disappeared (Figure 4). This gland consisted of cells containing many granules that were intensely stained with eosin, and acini were mostly negative for both PAS and Alcian blue (Figure 4), indicating that the gland was serous. This gland was connected to the vomeronasal lumen by some large secretory ducts (Figure 4) and did not open into the nasal cavity.

### 3.3. The Lumen and Epithelial Lining in Hedgehog VNO

The lumen was round-to-oval in the cross section throughout the VNO (Figure 2). The lumen was mostly lined by sensory epithelium in the rostral area (Figure 2B), and completely covered by non-sensory epithelium in the caudal area (Figure 2D). The medial and lateral halves in the middle of the lumen were respectively lined with sensory and non-sensory epithelia (Figure 2C).

**Figure 5:**
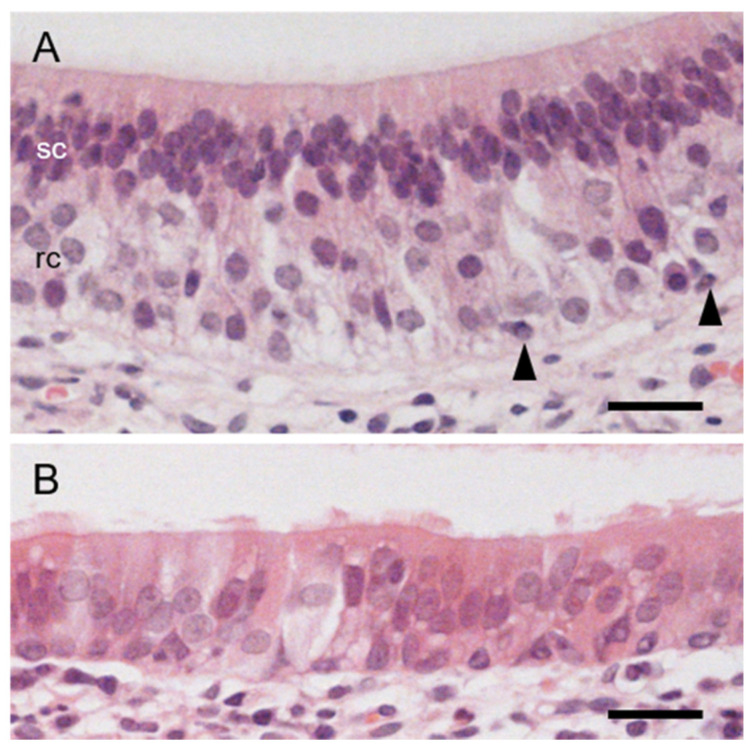
Histological features of sensory and non-sensory epithelia in hedgehog VNO. (**A**) Sensory epithelium. rc, nuclei of receptor cells; sc, nuclei of supporting cells. Arrowheads, basal cells. (**B**) Non-sensory epithelium. Hematoxylin-eosin stain. Bars, 20 μm.

Sensory epithelium was pseudostratified and comprised receptor, supporting and basal cells (Figure 5A). The nuclei of supporting cells were oval and densely arranged in the apical third, whereas those of receptor cells were round and located in the basal two-thirds of the sensory epithelium (Figure 5A). A few basal cells contacted the basal membrane (Figure 5A). Non-sensory epithelium was pseudostratified and contained ciliary and non-ciliary cells (Figure 5B).

**Figure 6:**
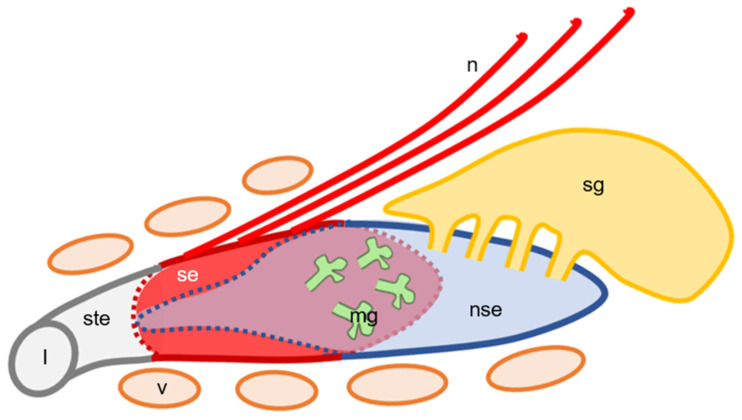
Schematic illustration of histological components of hedgehog VNO based on Figure 2. Left side, rostral; upper side, dorsal. l, lumen of VNO; mg, mucous glands; n, vomeronasal nerve bundles; nse, non-sensory epithelium; se, sensory epithelium; sg, serous gland; ste, stratified squamous epithelium; v, venous sinuses. In middle of VNO, sensory and non-sensory epithelia cover respectively medial and lateral walls of lumen.

The present findings of the hedgehog VNO, described above, are summarized as Figure 6.

## 4. Discussion

The histological features of the sensory epithelium in African pygmy hedgehog VNO are similar to those in other mammals that depend on the vomeronasal system [3]. This finding indicates that the hedgehog VNO is functional and plays important roles.

The hedgehog VNO connects to the incisive duct just before the nasal cavity. The VNO of most carnivores [14,15,16,17], ungulates [18,19], primates [20], elephants [21,22], hyraxes [23], and marsupials [24,25] is connected around the center of the incisive duct. The VNO in all these animals takes up substances through both the nasal and oral cavities, except in horses, in which the incisive ducts do not open to the oral cavity. On the other hand, the lumen of the VNO in rodents [26,27,28], rabbits [27,29], and armadillos [30] opens directly into the nasal cavity and does not connect to the oral cavity. The presence of VNO and its opening in bats depend on the species [31]. The orifice of the hedgehog VNO is intermediate between the two types described above, suggesting that the hedgehog uptakes odorants into the VNO mainly through the nasal cavity and sometimes through the oral cavity. Interestingly, the VNO of musk shrews, which belong to the same order (Eulipotyphla) as hedgehogs [8], opens into the nasal cavity directly [32]. Thus, the orifice of the VNO also depends on the species of Eulipotyphla, as in bats.

The vomeronasal capsule consists of cartilage in most mammalian species, including carnivores [15,16,17], ungulates [19,33,34,35], bats [31], primates [20], rabbits [27,29], elephants [21,22], hyraxes [23], armadillos [30], marsupials [24,25], and hedgehogs (revealed in the present study). However, the capsule is bony in some rodents, for example, rats, mice [27], hamsters [36], and caudal third in capybaras [37], although it is partly or mostly cartilaginous in mole rats [38]. Interestingly, the vomeronasal capsule of musk shrews also consists of bone [32], which we also confirmed [13], unlike that of the hedgehog.

The hedgehog VNO contains several small mucous glands and a well-developed serous gland. The VNO of brown bears also has two types of glands, but both are mucous [16]. Glands associated with the VNO in mammals are generally mucous [3], and the secretory substances form a mucous layer covering the sensory epithelium to dissolve the odorants. We previously found that these glands in Laurasiatheria species, except carnivores, are predominantly positive for Alcian blue [13], according to the findings of all 11 studied species including musk shrews. However, the glands located under the non-sensory epithelium of the hedgehog VNO, which seem to be corresponding glands, are PAS-positive and AB-negative. These findings suggest that the properties of the mucous fluid and the substances detected by the VNO differ between hedgehogs and shrews.

The most notable finding in this study is the well-developed serous gland associated with the hedgehog VNO. Reports indicated some serous-type cells in the mammalian vomeronasal glands [36,39,40], but as far as we can ascertain, well-developed serous glands related with VNOs have not been identified in any mammals. Serous secretions flow easily; thus the serous glands in the hedgehog VNO might flush out, rather than dissolve odorous substances. Among chemosensory organs, well-developed serous glands are gustatory glands associated with macroscopic papillae containing taste buds in the tongue [3]. They flush substances from the papilla and respond quickly to following stimuli. We speculate that the serous gland in the hedgehog VNO flushes out odorants to allow for an immediate response to subsequent stimuli.

The lumen of the VNO is round-to-oval throughout its entire length in the African pygmy hedgehog. It is also oval in common marmosets [41] and some species of mole rats [38], but is typically crescent-shaped in most mammals [3], including musk shrews [32]. The form of the duct within hedgehog VNO is more similar to that of large secretory gland ducts, and it might smooth the flow of serous gland secretions. In addition, venous sinuses that function as vasomotor pumps within the hedgehog VNO surround the entire lumen, although in VNOs with a crescent-shaped lumen, large veins are mostly located on the lateral side. Two mole rats with an oval VNO lumen, *Cryptomys hottentotus* and *Fukomys damarensis*, have venous sinuses that widely surround the lumen, whereas venous sinuses in *Heterocephalus glaber* mole rats with a crescent-shaped lumen are limited to the lateral region of the VNO [38]. These findings indicate that the venous sinuses need to surround the entire lumen to pump against the oval VNO lumen. This structural feature appears to be a disadvantage to the pumping function over a VNO with a crescent-shaped lumen and lateral venous sinuses. We speculate that flushing the oval VNO lumen with serous secretions to prepare for the next stimulus is more important for hedgehogs than efficient odorant uptake; that is, they might frequently use the VNO to recognize the external environment.

## 5. Conclusions

This is the first study to reveal the morphological features of the VNO in the African pygmy hedgehog. Unlike other mammals, the hedgehog has a well-developed serous vomeronasal gland in the VNO. The lumen of the hedgehog VNO is oval, like secretory gland ducts, and the venous sinuses around the lumen appear to pump against it. These morphological features seem to allow the removal of odorous substances from the VNO and might be favorable for receiving subsequent stimuli.

## Data Availability

All relevant data are within the manuscript, and are fully available without restriction.
